# Spatio-Temporal Change and Pollution Risk of Agricultural Soil Cadmium in a Rapidly Industrializing Area in the Yangtze Delta Region of China

**DOI:** 10.3390/ijerph15122743

**Published:** 2018-12-05

**Authors:** Xianghua Xu, Jiaying Qian, Enze Xie, Xuezheng Shi, Yongcun Zhao

**Affiliations:** 1 Jiangsu Key Laboratory of Agricultural Meteorology, Nanjing University of Information Science &Technology, Nanjing 210044, China; xuxianghua@nuist.edu.cn (X.X.); tomato12345@163.com (J.Q.); 2 State Key Laboratory of Soil and Sustainable Agriculture, Institute of Soil Science, Chinese Academy of Sciences, Nanjing 210008, China; ezxie@issas.ac.cn (E.X.); xzshi@issas.ac.cn (X.S.)

**Keywords:** agricultural soils, cadmium (Cd), potential pollution risk, industrialization and urbanization

## Abstract

The impacts of rapid industrialization on agricultural soil cadmium (Cd) accumulation and their potential risks have drawn major attention from the scientific community and decision-makers, due to the high toxicity of Cd to animals and humans. A total of 812 topsoil samples (0–20 cm) was collected from the southern parts of Jiangsu Province, China, in 2000 and 2015, respectively. Geostatistical ordinary kriging and conditional sequential Gaussian simulation were used to quantify the changes in spatial distributions and the potential risk of Cd pollution between the two sampling dates. Results showed that across the study area, the mean Cd concentrations increased from 0.110 mg/kg in 2000 to 0.196 mg/kg in 2015, representing an annual average increase of 5.73 μg/kg. Given a confidence level of 95%, areas with significantly-increased Cd covered approximately 12% of the study area. Areas with a potential risk of Cd pollution in 2000 only covered 0.009% of the study area, while this figure increased to 0.75% in 2015. In addition, the locally concentrating trend of soil Cd pollution risk was evident after 15 years. Although multiple factors contributed to this elevated Cd pollution risk, the primary reason can be attributed to the enhanced atmospheric deposition and industrial waste discharge resulting from rapid industrialization, and the quick increase of traffic and transportation associated with rapid urbanization.

## 1. Introduction

Cadmium (Cd) is a toxic heavy metal which occurs naturally in all soils [1]. As a non-essential element that negatively affects plant growth and development, Cd is much less mobile in soils than in air and water. However, the mobility of Cd in soils is high compared to the other heavy metals, and it can be taken up readily by plants [2], thereby enhancing the exposure risks of humans to Cd. Consequently, Cd is recognized as an extremely significant pollutant in agricultural soils.

Information on the spatial patterns of soil Cd is important, because it can serve as a basis for risk assessment, soil remediation, and effective management recommendations. Although large-scale spatial distribution patterns of soil Cd were mainly governed by natural factors such as geological setting and parent material [3,4], anthropogenic factors such as industrial activities [5,6], agricultural management practices [7,8], and the urbanization process [9,10] were often the primary reasons for the local-scale Cd anomaly patterns. For example, the spatial distribution patterns of Cd in agricultural soils of Europe were mainly governed by geology, whilst the natural anomaly patterns in several local areas were overprinted by anthropogenic emissions from former mining, ore processing and related metal industries [3]. In addition, some Cd anomalies in European agricultural soils can be attributed to urbanization and fertilization [3]. Spatial patterns and variation analysis of soil Cd in the Guangdong Province of China revealed that the overall spatial distributions of soil Cd were dominated by the parent material properties, while the accumulation of Cd in local soils were primarily caused by the enhanced Cd inputs from anthropogenic sources, for example, lead zinc ore exploitation, metal smelting, vehicle exhausts, and phosphate fertilization [4].

Cd was officially identified as a predominant metal pollutant in the agricultural soils of China [11]. Many studies have focused on the spatial distribution pattern and pollution risk of Cd in Chinese agricultural soils [6,12,13,14,15,16,17,18], but the spatio-temporal analysis using direct measurements over two sampling dates at a relatively large area remained unavailable, and only rather limited county-scale studies were reported, e.g., Li et al. [19].

The southern part of the Jiangsu Province is one of the most developed areas in the Yangtze River Delta region of China. The impacts of rapid industrialization, urbanization, and agricultural intensification on the spatio-temporal change of soil Cd levels in the area have drawn major attention from the scientific community and decision-makers [6,20,21]. Therefore, the specific objectives of this study were: (1) to explore the spatio-temporal changes of Cd levels in the agricultural soils over the past 15 years (2000 to 2015) and identify the areas with significant Cd accumulation over that time span; (2) to evaluate the potential risks of soil Cd pollution in the two time points, and discuss the primary reasons for the soil Cd accumulation.

## 2. Materials and Methods

### 2.1. Study Area

**Figure 1:**
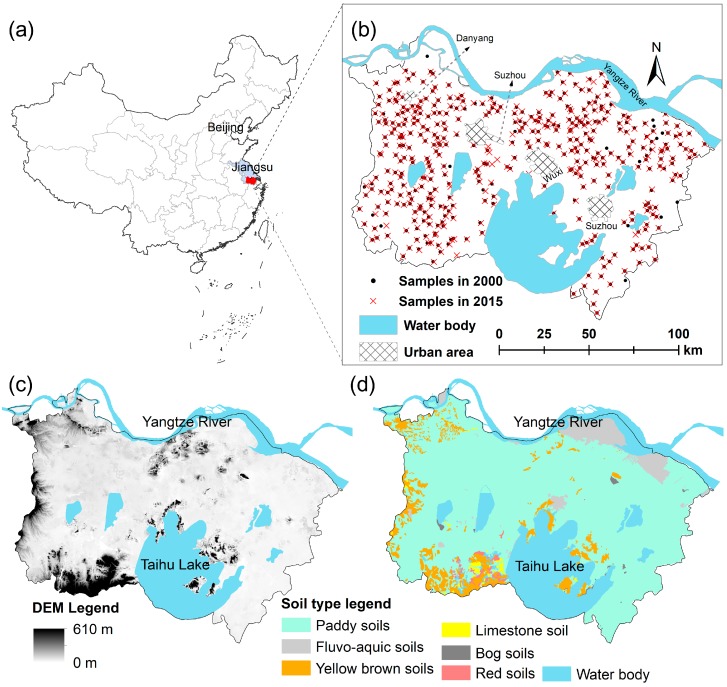
The location (**a**), soil sampling (**b**), topography (**c**), and soil distribution pattern (**d**) maps of the study area.

The study area is located in the southern part of the Jiangsu Province in the East China (Figure 1a), including three prefecture-level cities—Suzhou, Wuxi, and Changzhou—and a county-level city, Danyang (Figure 1b). The total land area of the study area is 18,703 km^2^ and is inhabited by a population of 22.8 million. The area has a subtropical monsoon climate, with a mean annual temperature of 15.6 °C and 900–1200 mm annual rainfall. The topography of the area is characterized by low mountains in the southwest and low plains in most parts of the study area (Figure 1c). The major soil types in the area are paddy soils (Stagnic Anthrosols), gray fluvo-aquic soils (Anthrostagnic-Dark-Cambosols), and yellow brown soils (Ferri-Udic Argosols). The soils along the Yangtze River, in the northern parts of the study area, are slightly alkaline grey fluvo-aquic soils that developed on the Yangtze River alluvium. Paddy soils derived from alluvial materials, lacustrine deposits, or loess are distributed in most parts of the study area, while yellow brown soils derived from residual and/or slope deposits are mainly located in the low-mountain areas (Figure 1d).

The study area, with thousands of years of paddy rice-planting history, is now a typical area dominated by industrial economy. The gross domestic product (GDP) from industrial outputs in both 2000 and 2015 accounts for ~50% of the total GDP in the area, while the proportions of GDPs from the agriculture sector decreased from ~6% in 2000 to ~2% in 2015.

### 2.2. Soil Sampling and Chemical Analysis

A total of 406 agricultural topsoil samples (0–20 cm) across the study area were collected in 2000 (Figure 1b). The sampling sites were determined by major soil types, land use, and a relatively even coverage of the whole study area. All of the sampling locations were recorded by hand-held GPS (Garmin, KS, USA), and the related background information, such as the soil type, topographic characteristics, and land use history, were also recorded in detail. The soil samples were air-dried, ground, and sieved to pass 100 meshes, and the total Cd in 2000 (denoted Cd2000 hereafter) were determined by using the graphite furnace AAS method [22].

In 2015, the sites sampled in 2000 were revisited and resampled (Figure 1b). However, because of the significant changes in land use (in particular the rapid expansions of residential industrial land during the period from 2000–2015) as well as the inherent locating errors of hand-held GPS, some of the sampling locations in 2015 were slightly different from those in 2000. The sampling sites in 2015 were chosen to: (1) match the sample sites of 2000 as closely as possible; (2) be on the identical soil types and topographic characteristics; and (3) have the most typical cropping system around the sites. Sample locations in 2015 were also recorded by the hand-held GPS, and the soil Cd concentrations in 2015 (denoted as Cd2015 hereafter) were determined using the same method as the sampling campaign in 2000.

### 2.3. Mapping the Spatio-Temporal Changes and Potential Risk of Cd Pollution

The spatial distribution patterns of soil Cd2000 and Cd2015 were first predicted using the Ordinary Kriging (OK) method. Then, the overall trends of changes in Cd spatial patterns over the time span 2000–2015 were estimated by subtracting the OK-predicted Cd2015 from the OK-predicted Cd2000. During the OK prediction process of Cd in the two sampling dates, the optimal models selected from the Spherical, Exponential, and Matern models were fitted to the experimental varograms of Cd2000 and Cd2015, respectively.

To quantify the uncertainty intervals of Cd changes that were derived from the subtraction of OK-predicted Cd2015 and Cd2000, 1000 times of sequential Gaussian simulations (SGS, see [23]) based on the variogram models of Cd2000 and Cd2015 were also conducted, respectively. The SGS employed in this study was conditional simulation, which means that the Cd values at the soil sampling locations were maintained in the SGS-generated simulation maps. The 1000 equiprobable simulation maps of Cd changes, derived from the random subtraction of the 1000 SGS-generated Cd2015 simulation maps and the 1000 SGS-generated Cd2000 simulation maps, were used to represent the uncertainty intervals of changes in Cd derived from the subtraction of OK-predicted Cd2015 and Cd2000.

The spatial distribution patterns of the soil Cd potential pollution risks in the two sampling dates were represented by the probability maps of Cd concentrations exceeding the background value 0.2 mg/kg (Chinese Environmental Quality Standard for Soils, GB 15618-1995), using the 1000 SGS-generated Cd2000 simulation maps and the 1000 SGS-generated Cd2015 simulation maps. The probability that unknown Cd concentration z(x) at location x exceeded the background value 0.2 mg/kg was calculated by Prob(z(x) > 0.2) = n(x)/1000, where n(x) is the number of simulation maps where simulated Cd value at location x exceeded 0.2.

## 3. Results

### 3.1. Descriptive Statistics and Spatial Variability of Cd

**Figure 2:**
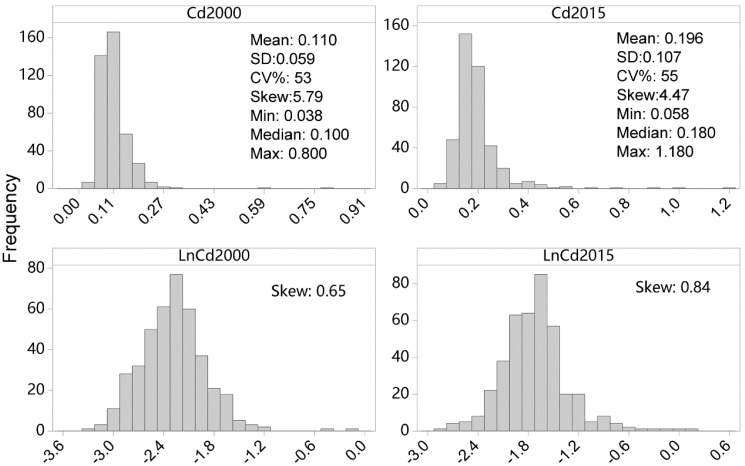
Histograms and summary statistics of topsoil Cd concentrations (mg/kg). Min, Max, SD, and CV represent minimum, maximum, standard deviation, and coefficient of variation, respectively.

Figure 2 presents the histograms and descriptive statistics of soil Cd concentrations from the two sampling dates. The descriptive statistics showed that the mean values of topsoil Cd concentrations in the study area increased from 0.110 mg/kg in 2000 to 0.196 mg/kg in 2015. The net increment of mean Cd concentrations was 0.086 mg/kg, equivalent to ~78% of the Cd2000 concentrations. The rate of Cd changes over that time period was estimated at 5.73 μg/kg/year, being slightly lower than the meta-analysis estimation (8.13 μg/kg/year) of the Yangtze River Delta region over the similar time span (2000–2014) by Shao, Zhan, Zhou and Zhu [6]. The median Cd2000 and Cd2015 concentrations were 0.100 and 0.180 mg/kg, respectively. The median values of the Cd concentrations from the two sampling dates were both significantly larger than the baseline median value of Chinese soils (0.079 mg/kg) provided by Wei et al. [24]. The ranges of Cd2000 and Cd2015 concentrations were estimated at 0.762 and 1.122 mg/kg, respectively. The coefficient of variation (CV) for Cd2000 and Cd2015 both exceeded 20%, indicating considerable variability of soil Cd in the two sampling dates. The value distributions of Cd2000 and Cd2015 are both strongly positively skewed, suggested that larger Cd concentration values existed and the spatial distribution of soil Cd in the two sampling dates are not homogeneous. Given that the coefficients of skew for soil Cd data in both sampling dates are all outside the interval [−1, 1], a natural logarithmic transformation was applied to the Cd data in the two dates, respectively, in order to eliminate the strongly positive skewness of the Cd distribution. The skew of Cd data decreased significantly after log transformation, and the Kolmogorov–Smirnov normal tests indicated that the value distribution of Cd2000 is normal (KS = 0.04, *p* > 0.05), while Cd2015 is approximately but not strictly normal distributed (KS = 0.09, *p* < 0.05).

**Figure 3:**
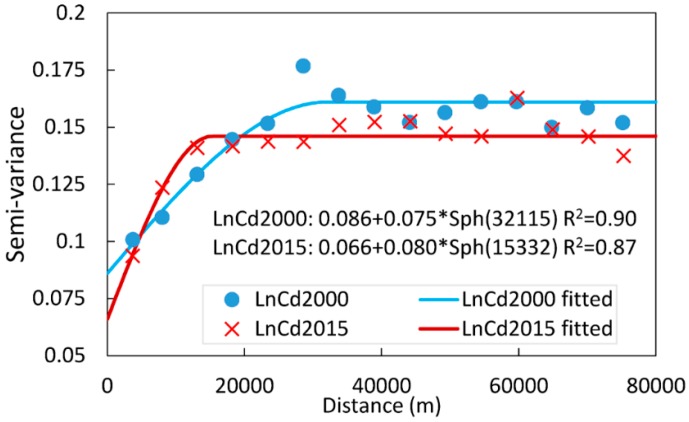
Experimental and fitted variograms of soil Cd (Cadmium) concentrations in the two sampling dates.

Since the skew of log-transformed Cd2015 is less than one, although its value distribution was not strictly normal, no further transformation was applied for the subsequent geostatistical analysis as suggested by Webster and Oliver [25]. The semivariance analysis showed that the soil Cd2000 and Cd2015 concentrations both exhibited spatial auto-correlative structures (Figure 3), indicating that they responded to processes that occur throughout the study area. The spatial dependencies, however, differed between the Cd concentrations in the two sampling dates, as illustrated by the nugget to sill ratios, as well as the ranges of spatial auto-correlation. The nugget to sill ratios for Cd2000 and Cd2015 were estimated at 53% and 45%, respectively, suggested that the spatial dependents of Cd in the two dates are both moderate, as the nugget to sill ratio was within 25–75%. The soil Cd2000 concentrations exhibited a relatively large auto-correlation range, with a significant spatial structure extending up to 32 km. Meanwhile, for Cd2015, a smaller auto-correlation range of 15 km can be intuitively observed, indicating that a smaller-scale spatial continuous process like local pollution diffusion may have stronger impacts on the Cd2015 spatial distribution.

### 3.2. Spatio-Temporal Patterns of Changes in Cd

**Figure 4:**
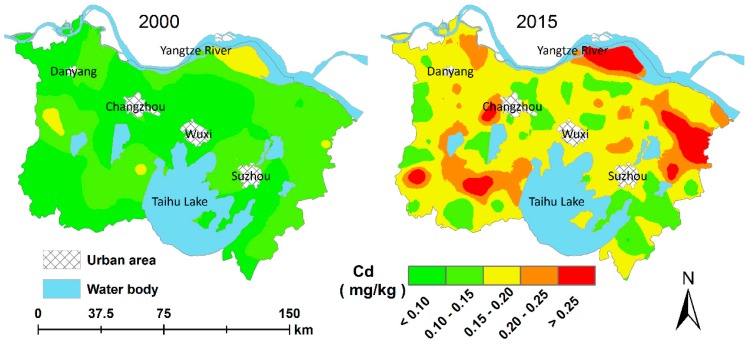
Ordinary kriging predicted spatial distribution maps of soil Cd (Cadmium) in 2000 and 2015.

The OK-predicted spatial distribution maps of Cd2000 and Cd2015 (Figure 4) demonstrate that the spatial patterns of Cd from the two sampling dates were significantly different. In 2000, the spatial patterns of Cd were relatively simple, with higher Cd values mainly distributed in the northern parts of the study area, lower values mainly in the southwestern parts and parts surrounding the large urban areas, while Cd concentrations in other parts of the study area were intermediate. However, in 2015, the spatial patterns of Cd were more complicated than those in 2000 due to a shorter spatial correlation range (~15 km, see Figure 3). More high-value patches of Cd can be intuitively observed in 2015, and high- and low-value patches of Cd were scattered across the whole study area (Figure 4).

**Figure 5:**
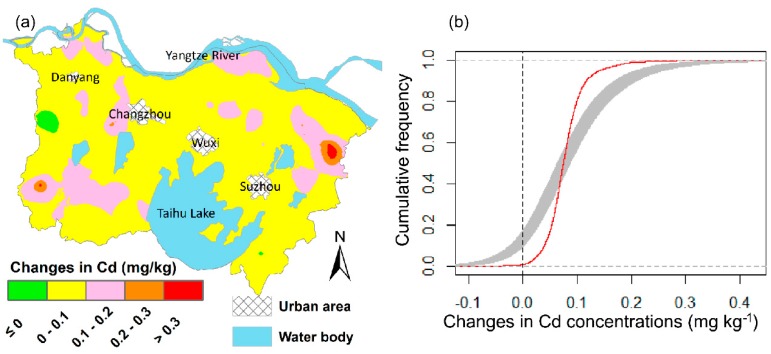
Spatial patterns (**a**) and cumulative frequency curves (**b**) of soil Cd change derived by ordinary kriging (shading area in Figure 5b is the uncertainty intervals of Cd changes).

The spatial distribution maps of changes in soil Cd concentrations derived from OK are presented in Figure 5a. A visual inspection of the Cd change map shows that the soil Cd concentrations in most parts of the study area increased over the past 15 years. Areas with Cd increment >0.2 mg/kg were mainly distributed near Suzhou city, and areas with Cd increments ranging from 0.1 to 0.2 mg/kg were scattered across the study area, while most parts of the study area had a Cd increment of less than 0.1 mg/kg. However, although the OK-predicted Cd maps are suitable for representing the overall trend of Cd spatial distribution patterns in the study area, using the subtraction of OK-predicted Cd2000 and Cd2015 maps to delineate the area of Cd changes may still be subject to large uncertainties, due to the smoothing effect inherent in the kriging interpolation method [25]. Figure 5b presents the cumulative frequency curve of Cd change derived from the OK2015–OK2000 method the (subtraction of OK-predicted Cd2015 and Cd2000, the solid red curve), and its associated uncertainty intervals (the shaded area) determined by random subtraction of SGS-generated Cd2000 and Cd2015 simulation maps. Most parts of the cumulative frequency curve for Cd changes derived from OK2015–OK2000 fell outside the uncertainty intervals of the method. In particular, a significant overestimation of areas with increased Cd concentrations over the past 15 years can be clearly observed, indicating a high uncertainty in detecting the Cd changes when using the direct subtraction between OK-predicted Cd maps from the two sampling dates.

**Figure 6:**
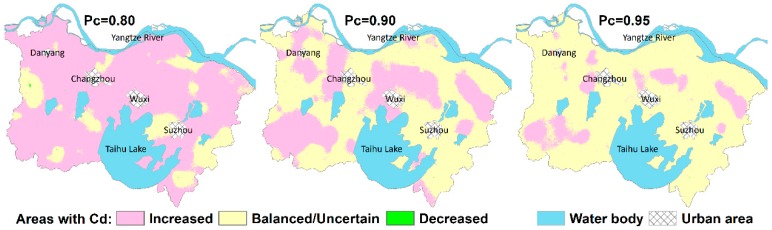
Areas with increased, decreased, and balanced (including uncertain) soil Cd (Cadmium) derived from different critical probabilities.

In order to identify the areas with significantly increased/decreased Cd while simultaneously quantifying their associated uncertainties, the probabilities of Cd2015 > (or <) Cd2000 were calculated through the random comparisons of SGS-generated Cd2015 and Cd2000 simulation maps. With a given critical probability Pc, the areas with Cd2015 > Cd2000 and Cd2015 < Cd2000 can be obtained based on the rule Prob (Cd2015 > Cd2000) ≥ Pc and Prob (Cd2015 < Cd2000) ≥ Pc, respectively. The areas with Prob (Cd2015 > Cd2000) < Pc or Prob (Cd2015 < Cd2000) < Pc can be considered as balanced in the meaning of changes in Cd being not significant at the given critical probability, or still uncertain due to measurement error and spatial variability that occurs over distances less than the shortest sampling interval. Here, a critical probability of 0.8, 0.9, and 0.95 was selected with an order of increasing confidence levels. Figure 6 shows that the identified areas where Cd accumulated significantly over the 15-year time span declined as the selected critical probability increased. The area percentages of Cd-increased area were estimated at 83.16%, 36.19%, and 12.24% for critical probabilities 0.80, 0.90, and 0.95, respectively. Meanwhile, areas where changes in Cd concentrations were not significant or uncertain covered 16.82%, 63.80%, and 87.76%, respectively, with the given critical probabilities of 0.8, 0.9, and 0.95, respectively. However, areas with decreased Cd over the past 15 years are almost negligible, accounting for only 0.02%, 0.005%, and <0.005%, respectively, with the given Pc of 0.80, 0.90, and 0.95, respectively. Overall, approximately 12% of the study area had significant Cd accumulation over the past 15 years, with a confidence level of 95%. The Cd-increased areas are mainly located in the northeast of Suzhou and Wuxi, southwest of Changzhou, and the southwestern parts of the study area.

### 3.3. Potential Risk of Soil Cd Pollution

**Figure 7:**
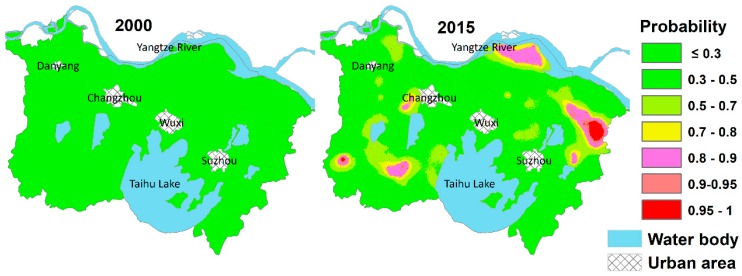
Probability maps of soil Cd (Cadmium) concentrations exceeding the background value.

The probability maps of potential Cd pollution risk are presented in Figure 7. The probability maps of Cd2000 exceeding the background value demonstrate that the areas with potential risk of Cd pollution in 2000 are extremely limited, and the area percentages with Cd exceeding the background value were only 0.009%. However, in 2015, the areas with high-probability pollution risk increased significantly. The area percentages with Cd2015 being greater than the background value at critical probabilities 0.8, 0.9, and 0.95 were 5.31%, 1.48%, and 0.75%, respectively. Locally, several areas with high probabilities of Cd2015 exceeding the background value 0.2 mg/kg are mainly located in the north and east of the study area. The areas where Cd2015 exceeded background value are obviously larger than those in 2000, indicating that the impacts of external Cd inputs on the elevated potential risk of Cd pollution are significant. Although the areas with high-probability potential pollution risk remained limited, the local concentrating trend of soil Cd pollution was evident.

## 4. Discussion

**Figure 8:**
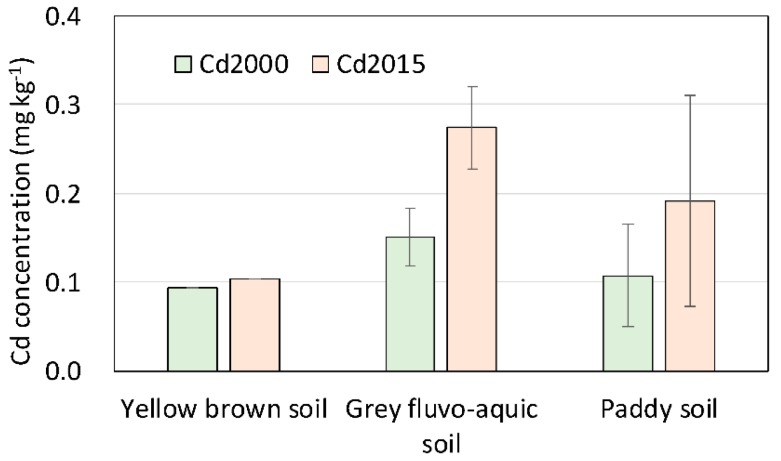
Soil Cd (Cadmium) concentrations in predominant soil types of the study area.

Cd in soils was derived from both natural and anthropogenic sources [1]. Soil types that differed in underlying bedrocks or transported parent materials had profound effects on soil Cd levels [26]. The soil Cd levels in the study area were closely related to the differences in soil types. Figure 8 shows that the mean Cd concentrations for the grey fluvo-aquic soils that developed on the Yangtze River alluvium were the highest among the three predominant soil types. The mean Cd levels for yellow brown soils were lowest, while for paddy soils, the mean Cd levels were intermediate. Although mean Cd levels in the three predominant soil types increased significantly over the past 15 years, the sorting orders of Cd levels for each of the soil types were still unchanged, suggesting that the influences of soil types on Cd levels remained important. For example, the Yangtze River alluvium as a transported parent material was one of the main natural sources of soil Cd, and the grey fluvo-aquic soils that developed on these alluviums were naturally enriched in Cd [27].

**Figure 9:**
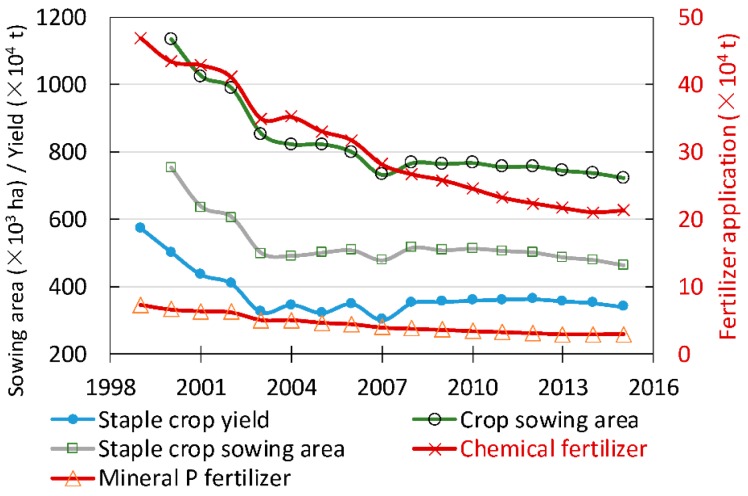
Changes in crop sowing area, crop yield, and amount of chemical fertilizer application since 1999 (data sourced from the Jiangsu Bureau of Statistics, http://tj.jiangsu.gov.cn/).

Agricultural activities accounted for ~63% of the total annual Cd inventory in Chinese agricultural soils [28], and the accumulation of Cd in agricultural soils of China was closely associated with the management practices of soil fertility [16]. Chemical fertilizer and manure application were important anthropogenic inputs of Cd to agricultural soils [8,29,30]. However, the application of farmyard manure in agricultural soils of the study area almost disappeared since 2000 [31] due to the rising costs of labor, transportation, manure composite and the decline in economic gains, compared to the chemical fertilization [32]. Another main source of Cd from agriculture was the use of mineral P fertilizers [29]. Frequent applications of P fertilizers may cause significant accumulations of Cd in agricultural soils [7]. However, the agricultural production in the area persistently declined after 2000 due to the rapid developments of the industrial economy and the urbanization process. Figure 9 showed that the crop (including staple crops) sowing area, staple crop yields, and the amounts of chemical fertilizer applications in the area declined significantly during the period from 2000–2015. The crop sowing area decreased from 1.13 Mha in 2000 to 0.72 Mha in 2015. In addition, as a predominant practice affecting Cd accumulations in agricultural soils, the application of mineral P fertilizer in the area reduced from 66,370 t (58.7 kg/ha) in 2000 to 29,840 t (41.3 kg/ha) in 2015 (Figure 9). This indicated that the use of Cd-containing fertilizers may not be the primary reason for the significant increase in the soil Cd concentrations of the area over the past 15 years, although the historical contributions of mineral P fertilizer application to the Cd accumulation in local soils of the areas should not be overlooked [21].

**Figure 10:**
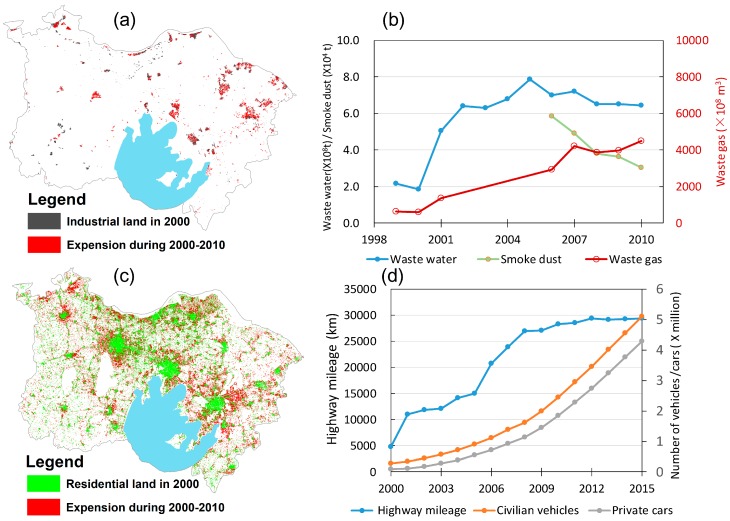
Changes in industrial and residential lands and the amounts of the industrial waste discharges over the period of 2000–2010, and the transportation-related information during 2000–2015 (land cover data were sourced from Land Cover Atlas of the People’s Republic of China (1:100,000), Land Cover Atlas of the People’s Republic of China Editorial Board [33], and other data were sourced from the Jiangsu Bureau of Statistics, http://tj.jiangsu.gov.cn).(**a**) changes in industrial land; (**b**) changes in residential land; (**c**) industrial waste discharge; (**d**) transportation-related changes.

Atmospheric deposition and industrial waste water discharge are also major anthropogenic factors governing the Cd levels in agricultural soils [3,6,12]. A significant expansion trend of industrial land during the period from 1999–2010 can be clearly observed from Figure 10a. The emissions of industrial waste gases and discharges of industrial waste waters also increased significantly over the same time period (Figure 10b). Industrial emissions of waste gases across the area increased by ~7 fold, from ~62 billion m^3^ in 1999 to ~440 billion m^3^ in 2010, while industrial waste water discharge increased from ~0.2 billion tons in 1999 to ~0.6 billion tons in 2010, suggesting that the significant accumulations of Cd in agricultural topsoils of the study area can be primarily attributed to the rapidly industrializing process. This was consistent with previous research identifying that atmospheric deposition originated from industrial coal combustion as the dominant influence on soil Cd enrichment in the Yangtze River Delta region [6]. Nonetheless, the expansions of residential land area over the period from 2000–2010 were also remarkable (Figure 10c), and the developments of traffic and transportation associated with rapid urbanization should not be overlooked. For example, highway mileages and civilian vehicles in the area increased by 6-fold and 17-fold, respectively, over the period from 2000–2015 (Figure 10d). This suggests that the quick development of traffic associated with rapid urbanization was also an important affecting factor in soil Cd accumulations.

## 5. Conclusions

A significant accumulation of Cd in major agricultural topsoils of the study area occurred over the period from 2000–2015. Overall, the soil Cd for approximately 12% of the study area increased significantly, with a confidence of 95%. The mean Cd concentrations increased from 0.110 mg/kg in 2000 to 0.196 mg/kg in 2015, representing an average increase of 5.73 μg/kg/year. Multiple factors contributed to this Cd accumulation, including soil types that differed in underlying bedrocks and parent materials, mineral P-fertilization history, and the rapid industrialization and urbanization process. However, the primary reason for the significant Cd accumulation can be attributed to enhanced atmospheric deposition and industrial waste discharges due to the rapid development of industrial economy, and the quick increase in traffic and transportation associated with the urbanization process.

Areas with a potential risk of Cd pollution in 2000 were extremely limited, only covering 0.009% of the study area. However, in 2015, this figure increased to 0.75% with a confidence level of 95%. Although the areas with a high-probability potential pollution risk were still limited after the 15-year rapid development, the locally concentrating trend of soil Cd pollution risk in the study area was evident. Moreover, Cd is much less mobile in neutral and alkaline soils. However, the soil pH value, one of the major factors governing Cd speciation and absorption in soils, has declined significantly in the area over the past three decades [34]. This may increase the availability of Cd to crops, thereby enhancing the exposure risks of humans to Cd. Consequently, effective management and control practices and powerful policies should be persistently applied in the area.
